# Comparison of the efficacy of one-stage revision surgery guided by precise pathogen diagnosis and conventional two-stage revision for chronic knee periprosthetic joint infection

**DOI:** 10.1186/s42836-025-00308-z

**Published:** 2025-05-30

**Authors:** Zhengwei Xiao, Jinyuan Zeng, Zeyu Zhang, Baijian Wu, Zihao Hong, Yufeng Guo, Chengguo Huang, Zida Huang, Zhaoyang Wu, Wenming Zhang, Xinyu Fang, Wenbo Li

**Affiliations:** 1 Department of Orthopaedics, The First Affiliated Hospital of Fujian Medical University, Fuzhou, 350005 China; 2 Orthopaedics and Hand Surgery, National Regional Medical Center, Binhai Campus of the First Affiliated Hospital, Fujian Medical University, Fuzhou, 350212 China; 3 Fujian Provincial Institute of Orthopaedics, the First Affiliated Hospital, Fujian Medical University, Fuzhou, 350005 China; 4 Fujian Orthopaedics Bone and Joint Disease and Sports Rehabilitation Clinical Medical Research Center, Fuzhou, 350005 China; 5 Department of Orthopaedic Surgery, Changtai County Hospital, Zhangzhou, 363999 China; 6 Department of Orthopaedic Surgery, Pingnan County Hospital, Ningde, 352300 China

**Keywords:** Chronic knee periprosthetic joint infection, Precise pathogen diagnosis, Combined pathogen diagnostic strategies, One-stage revision

## Abstract

**Aims:**

This study aimed to assess whether the clinical outcomes of one-stage revision surgery for chronic knee periprosthetic joint infection (kPJI), guided by precision pathogen diagnosis strategies, are non-inferior to those of conventional two-stage revision surgery.

**Methods:**

A retrospective analysis was conducted on chronic kPJI patients who underwent precision pathogen detection and revision arthroplasty at the First Affiliated Hospital of Fujian Medical University between January 2016 and September 2022. Clinical characteristics, pathogen detection rates, targeted antibiotic therapy, reinfection rates, and functional outcomes were compared between patients who underwent one-stage and two-stage revision surgeries.

**Results:**

Thirty patients who underwent one-stage revision surgery with pathogen detection through precision pathogen diagnosis strategies were included in this study and were matched with 30 patients who received two-stage revision surgery via propensity score matching (PSM). The baseline clinical characteristics did not significantly differ between the two groups. Utilizing our optimized pathogen detection protocol, successful pathogen identification was achieved in all cases across both groups. The median duration of intravenous antibiotic administration in the one-stage revision cohort was 16.5 (8.5,23.0) days, followed by a 6-week course of sequential oral antibiotics. Both the one-stage and two-stage revision groups had 3 cases of reinfection each, with no statistically significant difference in success rates between the groups (*P* > 0.999). Furthermore, no significant differences were found in the range of motion (ROM) (*P* = 0.332) or Knee Society score (KSS) (*P* = 0.117) between the one-stage and two-stage revision groups at the 2-year postoperative follow-up. The Kaplan‒Meier survival curves for prosthesis infection-free survival nearly overlapped, with no statistically significant differences between the two groups (*P* = 0.675).

**Conclusion:**

When pathogen identification is achieved through precision diagnostic strategies, the efficacy of one-stage revision surgery combined with targeted antibiotic therapy is comparable to that of two-stage revision surgery.

## Introduction

Periprosthetic joint infection (PJI) following total knee arthroplasty significantly compromises knee function and quality of life [1] and is considered a severe complication. Although the reported incidence of PJI ranges from only 0.5% to 2.0% in various studies, it is frequently cited as a primary cause of prosthesis revision surgery [1, 2].

For acute knee periprosthetic joint infection (kPJI), the combination of antibiotic therapy and debridement with implant retention (DAIR) is commonly employed in clinical practice. In contrast, for chronic PJI, two-stage revision surgery remains the standard treatment globally, with a success rate of over 85% [3–6]. Nevertheless, two-stage revision requires multiple surgeries, resulting in extended treatment periods, increased complications, and increased costs [7, 8]. Additionally, determining the appropriate duration of antibiotic usage and the optimal timing for prosthesis implantation continues to pose challenges for clinicians [9–12].

One-stage revision surgery has been increasingly applied in clinical practice, which reduces treatment duration and costs [13]. However, various studies still report a notable recurrence rate of infection after this procedure [3, 14, 15]. To increase treatment success rates, medical centers worldwide are improving one-stage revision protocols by refining surgical techniques and antibiotic administration methods. Nonetheless, as an infectious disease, targeted antibiotic therapy based on accurate pathogen identification remains crucial for controlling infection successfully [16]. Therefore, precise microbiological diagnosis, especially preoperative diagnosis, is essential for the success of one-stage revision [3, 14–16]. In clinical practice, several factors can result in false-negative pathogen cultures in chronic PJI patients, including improper sampling techniques, low bacterial counts due to biofilm formation, prior antibiotic use, improper specimen handling, insufficient culture time, and inappropriate culture conditions [17, 18]. These issues can lead to delays in treatment and an increased risk of failure.

To address the challenges associated with PJI, our institution has implemented a rigorous protocol for specimen collection and handling during the preoperative and intraoperative phases [19, 20]. We have optimized cultural conditions [21] and integrated advanced molecular diagnostic techniques, including polymerase chain reaction (PCR) and metagenomic next-generation sequencing (mNGS) [21]. These innovations form the basis of a comprehensive and precise pathogen diagnostic workflow. Leveraging this approach, we have employed one-stage revision surgery followed by targeted antibiotic therapy for patients with chronic knee PJI (kPJI) caused by well-characterized pathogens.

This study aims to determine whether the effectiveness of one-stage revision surgery, guided by accurate pathogen detection strategies, is non-inferior to that of traditional two-stage revision surgery. These findings could provide critical insights and evidence for refining clinical protocols, potentially positioning one-stage revision as a viable and effective treatment option for chronic PJI.

## Materials and methods

### Patient selection

This retrospective study adheres to the principles outlined in the Declaration of Helsinki. The inclusion criteria were as follows: (1) patients diagnosed with chronic PJI at our institution who underwent one-stage revision surgery, and (2) second-stage revision patients matched through 1:1 nearest neighbor propensity score matching (PSM).

The exclusion criteria were as follows: (1) patients who underwent one-stage revision without a preoperative positive culture or a definitive molecular diagnosis of the pathogen; (2) patients who underwent two-stage revision without a definitive microbiological diagnosis; and (3) patients with less than two years of follow-up or incomplete clinical data. The diagnosis of PJI was based on the diagnostic criteria established by the European Bone and Joint Infection Society (EBJIS) [22].

This study was approved by the Ethics Committee of the First Affiliated Hospital of Fujian Medical University (Approval No. [2015] 084–2). Informed consent was obtained from all patients.

### Optimized pathogen detection strategies

#### Specimen acquisition and processing

Joint fluid acquisition: For all suspected kPJI patients, joint aspiration was performed under sterile conditions with ultrasound guidance after a two-week antibiotic washout period. The procedure was conducted by experienced physicians without the use of local anesthesia.

Ultrasonic sonication fluid acquisition: The removed prosthesis was placed in a sterile container with approximately 400 mL of sterile saline. The mixture was subjected to ultrasonic sonication (Model: S150, Elma Company, Germany) at 40 Hz for 5 min. The resulting sonication mixture was centrifuged at 4000 rpm for 5 min, and the supernatant was discarded, after which the bacteria were concentrated for culture and molecular testing.

Tissue homogenate preparation: Suspected infected tissue samples were collected from five areas around the prosthesis. Each sample was cut into 0.5 cm^3^ tissue blocks and placed in culture containers with 3 ml of brain‒heart infusion (BHI) broth. After 15 min of vigorous vortexing, the samples were homogenized via an automated high-speed tissue homogenizer (Model: JXFSTPRP24; Jingxin Industrial, Shanghai, China) at 40 Hz for 60 to 90 s to ensure thorough dispersion and bacterial release [22].

#### Microbial cultivation

Routine culture: Liquid samples or tissue homogenates were inoculated onto Columbia blood agar plates (batch number: HBPM0153, Qingdao Haibo Biotechnology, China) and incubated under aerobic and anaerobic conditions to simulate different microbial growth environments. The remaining samples were inoculated into BACTEC Plus/F bottles (Chinese Medical Device Registration No. 20182400392; Becton–Dickinson, Franklin Lakes, NJ, USA) designed for both aerobic and anaerobic conditions. These bottles were incubated in a BACTEC 9050 automated blood culture system (FX400; Becton–Dickinson), which continuously monitors and records microbial growth.

#### Optimized culture conditions

1. Extended incubation: Cultures were incubated for 2–3 weeks based on preliminary clinical assessments. 2. Custom growth conditions: The temperature, humidity, and gas environments were adjusted to suit specific microbial requirements, such as obligate aerobes, microaerophiles, facultative anaerobes, and obligate anaerobes. 3. Selective media: Targeted media were chosen based on the nutritional needs of specific bacteria to improve pathogen detection rates. Examples include mannitol salt agar for *Staphylococcus aureus*, blood agar for Streptococcus species, Columbia agar with 5% sheep blood for coagulase-negative staphylococci, anaerobic agar for Cutibacterium acnes, liquid‒solid combined culture for Mycoplasma, and Löwenstein-Jensen medium for *Mycobacterium tuberculosis*.

#### Molecular diagnostic techniques

PCR: Two experienced PCR technicians performed PCR amplification on joint fluid, sonication fluid, and tissue homogenate samples via primers targeting common bacteria involved in bone and joint infections. The PCR products were analyzed via agarose gel electrophoresis. A clear band indicated successful amplification of the target DNA sequence. If no band or an unclear band was observed, the PCR was deemed invalid. In such cases, two independent laboratory technicians reviewed the results to ensure accuracy.

mNGS: Using specialized DNA extraction kits, DNA was purified from joint fluid, sonication fluid, and tissue homogenate samples. The process involved physical and chemical cell lysis, removal of proteins and other impurities, and DNA purification. The DNA was then fragmented into small pieces of approximately 200–300 bp via sonication or enzymatic digestion. These fragments were amplified via PCR, fixed onto the surface of nanoballs to create DNA nanoballs, and sequenced on the BGISEQ- 4500 platform (BGI).

#### Determination and handling of contaminant bacteria

The specific methodology follows our previously published studies [23], integrating bioinformatics filtering and clinical context to effectively differentiate pathogens from contaminants.

1. Negative Control Reference Setup: Each batch of samples includes sterile blood samples from healthy volunteers as negative controls. Microbial reads detected in the negative control are systematically subtracted from patient samples to eliminate exogenous or cross-contamination. 2. Background Microbial Filtering: Based on laboratory and clinical experience, common environmental or reagent-derived contaminants (e.g., Burkholderia, Ralstonia, and Delftia) are predefined. These microorganisms are classified as pathogens only if their relative abundance at the genus level (RAG) exceeds 80%, an empirically determined threshold to minimize false positives. 3. Stringent Pathogen Identification Criteria: A microorganism is classified as a pathogen only if it meets the following criteria: (1) Sequencing-mapped read number (SMRN) ≥ 3 and at least five times higher than in the negative control. (2) Bacterial RAG ≥ 30% (independent fungal reference standards apply). (3) Alternatively, species-specific criteria apply (e.g., Mycobacterium tuberculosis is considered clinically significant with a single detected read).

#### Optimized pathogen detection strategies

Our optimized pathogen detection strategies integrate sample acquisition, pathogen culture, and molecular diagnostics to enhance pathogen detection. Further details and the specific flowchart can be found in our team's previous publications [24].

1. Preoperative procedures: (1) Patient assessment: A detailed medical history was collected, physical exams were performed, and serological and imaging studies were conducted. (2) Sample acquisition: After two weeks of antibiotic washout, ultrasound-guided joint aspiration of synovial fluid was performed. This fluid is used for white blood cell analysis, pathogen culture, PCR, and mNGS.
2. Preliminary diagnosis: Evaluation: Combine initial culture and molecular diagnostic results with clinical and imaging data to assess for PJI. If PJI is suspected or the results are unclear, intraoperative testing should be performed for more information.
3. Intraoperative procedures: (1) Sample acquisition: Optimized techniques were used to acquire joint fluid, ultrasonic sonication fluid, and tissue homogenates. (2) Intraoperative testing: specific culture media and conditions should be applied, and additional molecular diagnostics should be performed.
4. Dynamic adjustment: Culturing conditions: Culture conditions were adjusted on the basis of preoperative and intraoperative molecular results. For culture-negative but molecularly positive cases, customize culture conditions to improve detection rates. Reassess and retest for rare pathogens as needed. This streamlined approach aims to improve pathogen detection and enhance diagnostic accuracy in PJI patients.

### Surgical procedure and antibiotic protocol

1. Surgery: The surgeries were conducted by a senior chief surgeon under general or epidural anesthesia via an anterior approach. Patients with preoperative pathogen identification and sensitivity status received intravenous sensitive antibiotics 30 min before surgery. The other patients were administered 1 g of vancomycin and 1 g of ceftazidime intravenously 30 min before surgery. The procedure involved prosthesis removal, thorough debridement, and the implantation of a new prosthesis or spacer. For one-stage revisions, a new knee prosthesis was implanted with antibiotic-loaded bone cement (2 g of vancomycin and 1 g of meropenem per 40 g of cement) to ensure broad-spectrum coverage. Additional bone cement and spacers were applied as needed based on intraoperative bone loss. Two-stage revisions followed our previously reported procedure [25]. No intra-articular antibiotic injection or irrigation was performed during any of the procedures.
2. Targeted antibiotic therapy based on pathogen diagnosis: For patients with positive cultures, antibiotics were selected based on susceptibility results or pathogen-specific characteristics. If only molecular diagnostics were positive, a multidisciplinary team (MDT) of senior microbiologists and pharmacists guided the antibiotic choice, with adjustments made based on treatment response. For cases where both cultures and molecular diagnostics were negative, empirical antibiotics were used. Postoperatively, all patients received intravenous antibiotics for 2–6 weeks, followed by oral antibiotics with high bioavailability. The total treatment duration was 6 to 8 weeks, with adjustments based on inflammatory markers.

### Follow-up

Each group of patients underwent outpatient follow-up at 1 month, 3 months, 6 months, 1 year, and annually thereafter. If any infection-related clinical symptoms occurred, patients were instructed to return to the hospital for evaluation and treatment. We collected demographic data, preoperative and final follow-up inflammatory marker data (CBC, CRP, ESR), X-ray images, range of motion (ROM) data, Knee Society score (KSS), and reinfection rates. The primary outcome was the treatment success rate, defined as the absence of reoperation due to infection. Secondary endpoints included functional outcomes (assessed by ROM and the KSS) and inflammatory marker normalization (CRP < 10 mg/L, ESR < 30 mm/h). Infection control was determined based on the absence of infection-related symptoms (well-healed incision, no sinus tract formation, no pain during movement or at rest, no swelling, normal body and local temperatures), normal inflammatory markers, and imaging showing no signs of bone resorption or osteolysis. Treatment failure was defined as infection recurrence, reoperation, or death related to PJI. The criteria for diagnosing infection recurrence followed the 2011 PJI guidelines of the Musculoskeletal Infection Society (MSIS) [26]. For survival analysis, the observation time was calculated monthly, with infection recurrence or reoperation for debridement as the event outcome. Survival time was measured from the date of surgery to the date of the event diagnosis. At the last follow-up, patients who had not experienced reinfection or who died from non-PJI causes were considered censored data.

### Statistical analysis

The data were analyzed via SPSS 26.0 (SPSS Inc., Chicago, IL, USA). For normally distributed continuous variables, the data are presented as the means ± standard deviations (x̄ ± s), and comparisons within and between groups were performed via *t*-tests. For non-normally distributed continuous variables, data are presented as medians (interquartile ranges) [medians (IQRs)], and comparisons between groups were made via the Wilcoxon rank-sum test or the Mann‒Whitney U nonparametric test. Categorical variables are presented as frequencies and percentages (n, %) and were analyzed with the chi-square test or Fisher’s exact test, as appropriate. Survival analysis was conducted via the Kaplan–Meier method to plot survival curves, and differences in prosthesis infection-free survival rates were evaluated via the log-rank test. Statistical significance was set at *P* < 0.05.

## Results

### Clinical characteristics of the included patients

From January 2016 to September 2022, a total of 108 patients underwent total knee arthroplasty revision. After those with insufficient follow-up, those lost to follow-up, patients who did not receive a second-stage prosthesis after spacer placement, and second-stage revision patients without a definitive pathogen diagnosis were excluded, 30 one-stage revision patients were included. Using nearest neighbor PSM scoring, these patients were matched 1:1 with 30 corresponding two-stage revision patients. As shown in Table 1, the clinical characteristics of the two groups were analyzed, and the results revealed no statistically significant differences. The median age of the entire cohort of patients was 67.50 years (63.00, 71.75). All patients had a follow-up period of more than 2 years post-revision. A total of 21 patients (35%) had a sinus tract communicating with the joint. One patient in both the one-stage and two-stage revision groups died from causes unrelated to PJI.

**Table 1:** Baseline comparison of patients undergoing one-stage and two-stage revision surgery

	**Total** *n* = 60	**One-stag**e *n* = 30	**Two-stage** *n* = 30	***X***^***2***^***/t/Z***	***P***
Age, yrs(IQR)	67.50(63.00,71.75)	67.50(64.00,72.00)	67.5(61.00,71.50)	–0.481	0.630^a^
Sex, *n* (%)				1.364	0.243^b^
Male	16 (26.7)	10 (33.3)	6 (20.0)		
Female	44 (73.3)	20 (66.7)	24 (80.0)		
BMI, kg/m^2^(SD)	24.45(3.53)	25.10(4.10)	26.03(3.26)	–0.647	0.520^c^
Hypertension, *n* (%)	25 (41.7)	13 (43.3)	12 (40.0)	0.069	0.793^b^
Diabetes, *n* (%)	19 (31.7)	8 (26.7)	11 (36.7)	0.693	0.405^b^
Other comorbidities, *n* (%)	14 (23.3)	5 (16.7)	9 (64.3)	1.491	0.222^b^
Affected side, *n* (%)				0.268	0.605^b^
Left	28 (46.7)	17 (56.7)	15 (50.0)		
Right	32 (53.3)	13 (43.3)	15 (50.0)		
Sinus, *n* (%)	21 (35.0)	9 (30.0)	12 (40.0)	0.659	0.417^b^
Time from primary surgery to revision, mos. (IQR)	28.45(10.33,49.63)	23.12(9.87,45.72)	35.78(10.03,67.68)	–1.220	0.223^a^
WBC, × 10^9^/L (IQR)	6.67(5.39,8.72)	6.70(5.71,8.87)	6.63(5.03,8.07)	–0.402	0.688^a^
CRP, mg/L (IQR)	26.52(13.53,54.42))	30.70(19.14,64.24)	37.29(34.68)	–1.026	0.305^a^
ESR, mm/h (SD)	73.73(29.22)	70.79(27.58)	76.57(30.93)	–0.756	0.453^c^
SF-WBC, × 10^6^/L (IQR)	36,022.50(15,968.00,68,699.50)	45,315.00(25,193.00,68,082.50)	35,383.00(13,422.25,94,573.25)	–0.931	0.352^a^
SF-PMN, (IQR)	89.60 (83.60,93.20)	90.00(87.60,94.10)	88.80(79.58,91.35)	–2.047	0.041^a^
Duration of intravenous antibiotic therapy, days (IQR)	16.50(12.25,23.00)	16.50(8.50,23.00)	17.50(13.00,25.25)	–0.770	0.441^a^

*SD* standard deviation, *BMI* body mass index, *CRP* C-reactive protein, *ESR* erythrocyte sedimentation rate, *SF* synovial fluid, *WBC* white blood cell, *PMN* polymorphonuclear leucocyte

^a^Mann‒Whitney U nonparametric test

^b^Chi-square test

^c^Independent-samples *t*-test

### Pathogen detection results

In the cohort of patients who underwent one-stage revision, pathogen information was obtained from all individuals prior to surgery. Among these patients, 7 (23.3%) presented with negative conventional cultures but positive results from molecular diagnostics; following intraoperative culture optimization, all subsequently yielded positive cultures. For the two-stage revision patients, 7 cases (23.3%) lacked pathogen information from both conventional cultures and molecular diagnostics preoperatively, whereas 5 cases (16.7%) had negative conventional cultures but tested positive via molecular diagnostics. Ultimately, through optimized intraoperative microbiological detection processes, all patients in the two-stage revision group achieved pathogen identification, including 3 patients (10%) with negative conventional cultures but positive molecular diagnostic results. The details of pathogen detection can be found in Table 2.

**Table 2:** Pathogen detection rate

	**Total** *n* = 60	**One-stage** *n* = 30	**Two-stage** *n* = 30	***X***^***2***^	***P***
Preoperative culture detection rate, *n* (%)	41 (68.3)	23 (76.7)	18 (60.0)	2.132	0.094*
Preoperative molecular diagnosis detection rate, *n* (%)	49 (81.7)	26 (86.7)	23 (76.7)	0.316	0.684*
Intraoperative culture detection rate, *n* (%)	49 (81.7)	24 (80.0)	25 (83.3)	0.107	0.686*
Intraoperative optimized culture detection rate, *n* (%)	60 (100.0)	30 (100.0)	30 (100.0)	2.684	> 0.999*

^*^Chi-square test

**Fig. 1:**
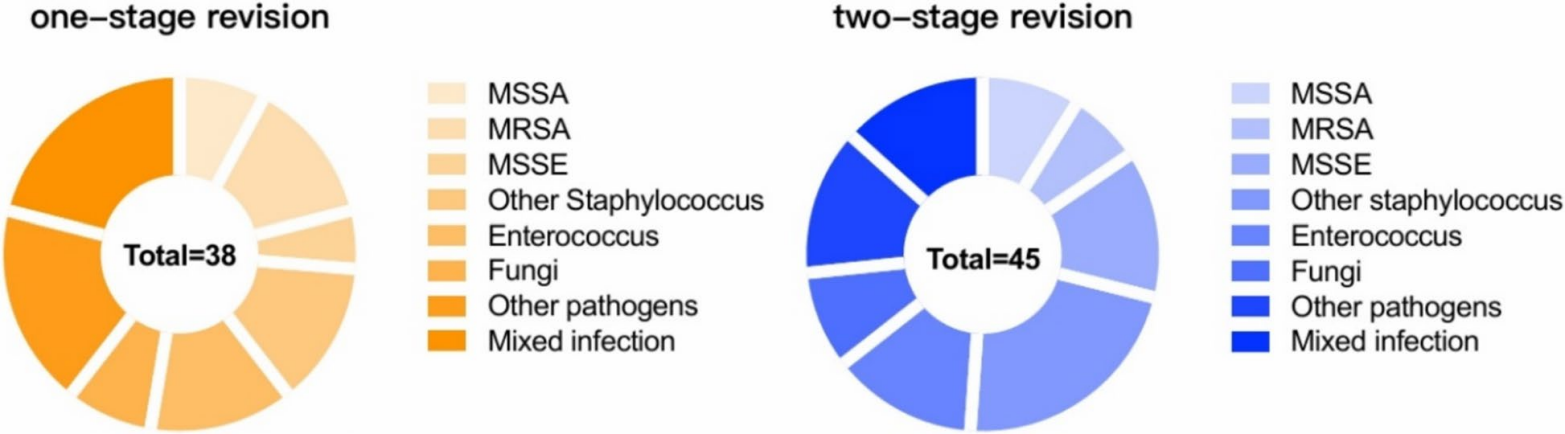
Pathogen detection results. MSSA: methicillin-susceptible Staphylococcus aureus; MRSA: methicillin-resistant Staphylococcus aureus; MSSE: methicillin-susceptible Staphylococcus epidermidis

The pathogen infection profile is shown in Fig. 1. The prevalence of mixed infections involving two or more microorganisms was 16.7% (5/30) among the one-stage patients and 13.3% (4/30) among the two-stage revision patients. Notably, in the one-stage revision cohort, 1 case of fungal infection and 2 cases of atypical pathogen infections were documented, specifically involving nontuberculous mycobacterium (Mycobacterium abscessus) and Rickettsial infections. In contrast, the two-stage revision cohort included 2 patients with fungal infections and 1 patient with an atypical pathogen infection, specifically a *Mycoplasma* infection.

### Targeted antibiotic therapy

All patients initially received empirical broad-spectrum antibiotics, typically vancomycin combined with a third-generation cephalosporin or quinolones combined with a third-generation cephalosporin. Upon the results of pathogen detection, antibiotics were adjusted according to susceptibility testing. In the one-stage revision group, 1 patient who was diagnosed with fungal infection through optimized cultures received antifungal treatment. One patient with NTM infection was treated with clarithromycin, rifampin, and ethambutol, whereas another patient with Rickettsia infection received doxycycline and moxifloxacin. In the two-stage revision group, 2 patients with fungal infections were treated with antifungal agents, and 1 patient with Mycoplasma infection was treated with doxycycline and moxifloxacin. The intravenous antibiotic regimens for both groups are shown in Table 3. The median duration of postoperative intravenous antibiotic use for patients in the one-stage revision group was 16.5 (8.5,23.0) days. Two patients (6.7%) in the one-stage group and 1 patient (3.3%) in the two-stage group required intravenous antibiotics for more than 6 weeks after their final surgery. Following intravenous therapy, all patients transitioned to oral antibiotics with high sensitivity and bioavailability for at least 6 weeks, with discontinuation based on clinical signs, symptoms, and inflammatory markers.

**Table 3:** Intravenous Antibiotic Regimen

	**One-stage** *n* = 30	**Two-stage** *n* = 30	***P***
Intravenous Antibiotic Regimen			0.666
Vancomycin + Ceftazidime	14	16	
Fluoroquinolone + Ceftazidime	3	2	
Vancomycin + Meropenem	4	5	
Cefazolin + Meropenem	6	4	
Antifungal drugs	1	2	
Special anti-infective regimens	2	1	
Addition of Rifampin	4	3	
Duration of intravenous antibiotic therapy, days (IQR)	16.5(8.5,23.0)	17.5 (13.0,25.3)	0.441

### Comparison of outcomes

**Fig. 2:**
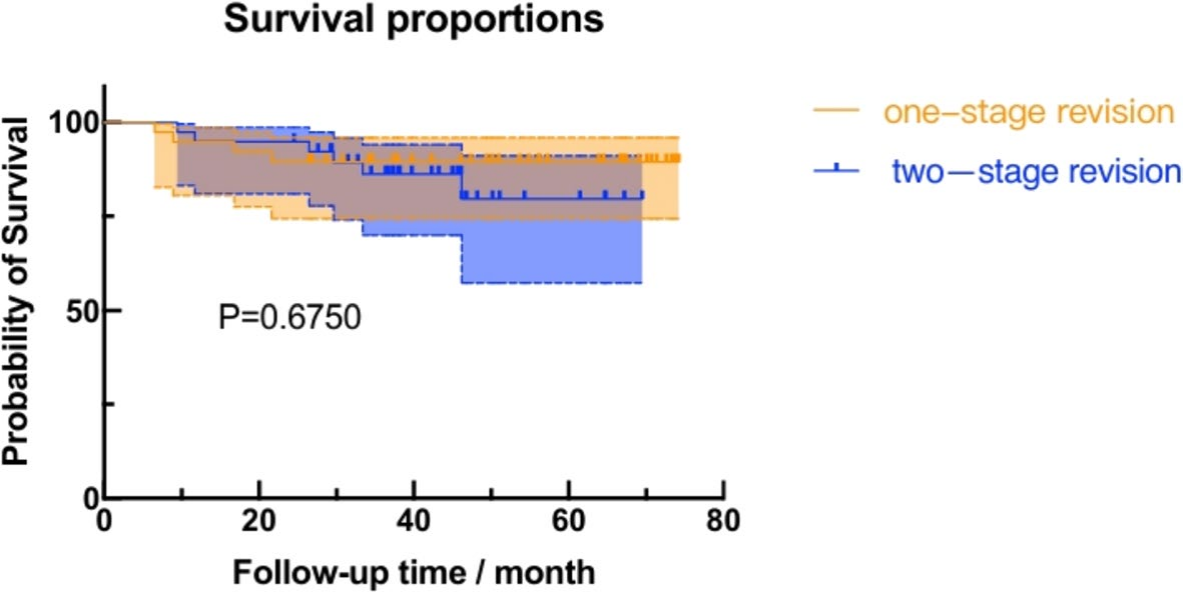
Kaplan–Meier survival curve analysis results for the two groups

Among the patients who underwent one-stage or two-stage revision, 3 patients in each group reported reinfection requiring further surgical intervention. The success rates were identical between the two groups: 90.0% (27/30) in the one-stage group vs. 90.0% (27/30) in the two-stage group (*P* > 0.999, Fisher's exact test). The Kaplan‒Meier survival curves for both cohorts are presented in Fig. 2 and demonstrate comparable survival rates (*P* = 0.675). The log-rank test results indicated that the prosthesis-free survival rate without infection in the one-stage revision cohort was not inferior to that in the two-stage revision cohort. As shown in Table 4, both groups of patients demonstrated significant improvements in ROM and KSS score postoperatively compared with the preoperative values (*P* < 0.05). Furthermore, there was no statistically significant difference in the ROM (*P* = 0.332) or KSS (*P* = 0.117) between the one-stage revision cohort and the two-stage revision cohort at the 2-year postoperative follow-up.

**Table 4:** Comparison of outcomes between one-stage and two-stage revision patients

**Groups**	**ROM (SD)**		*P*	**KSS (SD)**		*P*	**Reinfection**
	Preoperative	Postoperative		Preoperative	Postoperative		
**One-stage**	63.33(21.70)	93.07(17.73)	< 0.01†	50.50(7.54)	81.43(10.01)	< 0.01†	3
**Two-stage**	66.50(19.96)	88.50(18.44)	< 0.01†	49.00(8.12)	85.13(7.85)	< 0.01†	3
*P*	0.559†	0.332†		0.461†	0.117†		> 0.999‡

*ROM* range of motion, *KSS* Knee Society score, *SD* standard deviation

^†^Independent-samples *t*-test

^‡^ Fisher’s exact test

Among the 3 patients who experienced reinfection following one-stage revision, one had mixed infection with fungi, another had methicillin-resistant *Staphylococcus aureus* (MRSA), and the third had *Enterococcus faecalis*. Among the patients with reinfection in the two-stage revision cohort, one had a mixed infection, another had a fungal infection, and the third had methicillin-resistant coagulase-negative Staphylococcus. All reinfected patients underwent standard second-stage revision surgery.

## Discussion

Precise pathogen identification is essential for successful revision surgery in chronic PJI [16, 27]. By integrating and optimizing existing detection methods, our team significantly increased pathogen detection rates [19, 24, 28–30]. Guided by these optimized pathogen detection strategies, patients with confirmed pathogens in chronic kPJI patients who underwent one-stage revision surgery achieved outcomes that were non-inferior to those of patients who underwent two-stage revision surgery, offering valuable insights for clinical treatment.

Recent efforts to optimize treatment for chronic kPJI have led to comparisons between one-stage and two-stage revision surgeries. In a systematic review on chronic knee PJI, Pangaud et al. [13] reported that one-stage revision achieved an average eradication rate of 87.1%, whereas two-stage revision had an eradication rate of 84.8%. Both approaches yielded comparable outcomes in terms of infection eradication, functional improvement, and patient satisfaction. Another meta-analysis [15] suggested that one-stage revision might result in a lower reinfection rate than the two-stage approach does, offering a viable alternative for managing chronic kPJI. The success of PJI treatment depends on precise pathogen identification, thorough surgical debridement, and targeted antibiotic therapy. While most studies on one-stage revision focus on refining surgical techniques, our research emphasizes pathogen detection. We have made significant improvements in sample acquisition processes, culture optimization, and molecular diagnostics. Furthermore, we continuously adapt the culture conditions based on molecular diagnostic findings to achieve optimal detection of pathogens [19, 24, 28–30].

Our team utilizes ultrasound-guided sterile joint aspiration for preoperative synovial fluid sampling. Compared with conventional aspiration, this method allows precise needle placement into the effusion area, minimizing neurovascular injury while increasing accuracy, safety, and success rates. During intraoperative sample acquisition, we employ ultrasonic disruption of the prosthesis to obtain lysate for culture, particularly for biofilm-forming pathogens, significantly increasing the detection success rate [24, 30, 31]. For suspected intracellular pathogens such as *Legionella pneumophila*, *Mycobacterium tuberculosis*, and *Staphylococcus aureus*, where free pathogens in joint fluid are rare, we use tissue homogenization to release and isolate intracellular bacteria for more effective identification [19, 24]. We optimized pathogen detection by integrating various culture conditions tailored to common pathogens in osteoarticular infections, including extended culture times, precise environmental settings, and selective media [19, 21, 24, 28, 29]. Additionally, we employ PCR and mNGS for unbiased, rapid, and specific genetic detection of pathogens, independent of culture [16, 24, 28, 32]. The pivotal step involves providing timely feedback and implementing targeted adjustments on the basis of molecular diagnostic results. This process enables the application of individualized optimization strategies for suspected positive detections and allows for the validation of molecular findings by refining culture conditions to confirm their accuracy.

In one-stage revision surgeries, our team meticulously selected patients who had preoperative pathogen information or positive molecular diagnostic results, facilitated by optimized pathogen detection strategies. Notably, for the matched two-stage revision cases, pathogen information was ultimately obtained, even though some patients did not have definitive pathogen diagnoses preoperatively. Our findings indicate that, guided by this approach, one-stage revision surgery yielded non-inferior benefits to two-stage revision in terms of reinfection rates, postoperative joint range of motion, and joint function scores. Even in complex cases involving fungal infections, unusual pathogens, or the presence of sinus tracts, satisfactory outcomes were achievable with one-stage revision when guided by our optimized pathogen detection strategies. Consequently, we recommend employing these strategies for one-stage revision in chronic PJI patients to maximize success rates.

In the cohort of patients who underwent one-stage revision, we encountered two cases of atypical infections: one involving Mycobacterium abscessus and the other associated with Rickettsial infection. These infections present with nonspecific clinical symptoms, making them difficult to distinguish from common infections, and conventional culture conditions are inadequate for their isolation [33–35]. Furthermore, standard antibiotics or antituberculosis treatments are generally ineffective [35, 36]. Despite preoperative cultures being negative, the use of preoperative mNGS results allowed for the optimization of intraoperative sampling and postoperative culture conditions. This approach ultimately results in positive cultures and sensitivity profiles, leading to successful outcomes with one-stage revision surgery and tailored postoperative treatment. However, for cases with unclear pathogen diagnosis, two-stage revision arthroplasty remains a reliable option.

This study has several limitations: (1) As a single-center, retrospective study, it is prone to selection bias and has a limited sample size, which may affect the accuracy of the findings; (2) the exclusion of two-stage revision cases that did not proceed to the second stage may have resulted in an overestimation of the reinfection rate for two-stage revisions; (3) the follow-up period was relatively brief, necessitating extended follow-up and further case accumulation to increase the robustness of the results; and (4) This study exclusively included patients with definitive pathogen identification. The exclusion of culture-negative PJI may introduce selection bias and limit the generalizability of the conclusions. Future multicenter studies with larger sample sizes are needed to validate these findings.

## Conclusion

In summary, the optimized pathogen detection strategies employed in this study significantly increased pathogen detection rates in chronic kPJI patients. One-stage revision surgery guided by precise pathogen diagnosis combined with targeted antibiotic therapy has demonstrated efficacy that is non-inferior to that of two-stage revision surgery, establishing it as an effective treatment strategy for chronic kPJI.

## Data Availability

The data that support the findings of this study are available from the corresponding author upon reasonable request.
